# *N*,*N*′-Bis(2-amino­phen­yl)-3,4-diphenyl­thio­phene-2,5-dicarboxamide acetonitrile solvate

**DOI:** 10.1107/S1600536810008780

**Published:** 2010-03-13

**Authors:** Rizvan K. Askerov, Vladimir V. Roznyatovsky, Evgeny A. Katayev, Abel M. Maharramov, Victor N. Khrustalev

**Affiliations:** a Baku State University, Z. Khalilov St 23, Baku AZ-1148, Azerbaijan; b Chemistry Department, M. V. Lomonosov Moscow State University, Leninskie gory 1/3, Moscow 119991, Russian Federation; c A. N. Nesmeyanov Institute of Organoelement Compounds, Russian Academy of Sciences, Vavilov St 28, B-334, Moscow 119991, Russian Federation

## Abstract

In the title solvate, C_30_H_24_N_4_O_2_S·CH_3_CN, the substituted thiophene possesses approximate *C_s_*(*m*) intrinsic symmetry, with the mirror plane passing through the S atom and the mid-point of the (Ph)C—C(Ph) bond. Despite the main backbone of the mol­ecule being a long chain of conjugated bonds, it adopts a non-planar conformation due to the presence of various intra- and inter­molecular hydrogen bonds. The hydrogen bonds result in twist configurations for both the amido and amino­phenyl fragments relative to the central thio­phene ring. There are two intra­molecular N_amine_—H⋯O hydrogen bonds within the thio­phene-2,5-dicarboxamide mol­ecule that form seven-membered rings. In the crystal, the thio­phene-2,5-dicarboxamide mol­ecules form inversion dimers by four amide–amine N—H⋯N hydrogen bonds. The dimers are further linked into layers propagating in (100) both directly (*via* N_amine_—H⋯O hydrogen bonds) and through the acetonitrile solvate mol­ecules (*via* amine–cyano N—H⋯N and C_Me_—H⋯O inter­actions).

## Related literature

For general background to aromatic diamide diamines, see: Picard *et al.* (2001 ▶); Schneider & Yatsimirsky (2008 ▶). For related compounds, see: Sessler *et al.* (2005*a* ▶,*b* ▶), Katayev *et al.* (2007 ▶); Askerov *et al.* (2010 ▶).
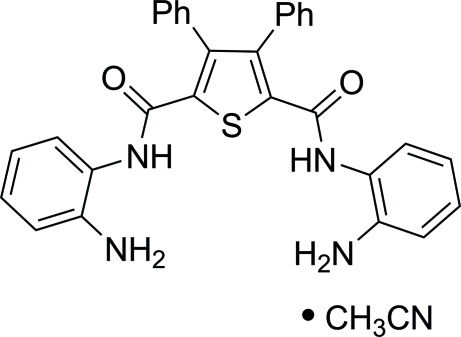


## Experimental

### 

#### Crystal data

C_30_H_24_N_4_O_2_S·C_2_H_3_N
*M* *_r_* = 545.65
Triclinic, 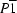

*a* = 9.0314 (9) Å
*b* = 11.5470 (11) Å
*c* = 13.0140 (12) Å
α = 93.206 (2)°
β = 92.504 (2)°
γ = 90.017 (2)°
*V* = 1353.7 (2) Å^3^
*Z* = 2
Mo *K*α radiation
μ = 0.16 mm^−1^
*T* = 120 K
0.24 × 0.21 × 0.18 mm

#### Data collection

Bruker SMART 1K CCD diffractometer
Absorption correction: multi-scan (*SADABS*; Sheldrick, 1998 ▶) *T*_min_ = 0.965, *T*_max_ = 0.972
13964 measured reflections
6491 independent reflections
5153 reflections with *I* > 2σ(*I*)
*R*_int_ = 0.018

#### Refinement

*R*[*F* ^2^ > 2σ(*F* ^2^)] = 0.048
*wR*(*F* ^2^) = 0.122
*S* = 1.01
6491 reflections
362 parameters
H-atom parameters constrained
Δρ_max_ = 0.40 e Å^−3^
Δρ_min_ = −0.26 e Å^−3^

### 

Data collection: *SMART* (Bruker, 1998 ▶); cell refinement: *SAINT-Plus* (Bruker, 1998 ▶); data reduction: *SAINT-Plus*; program(s) used to solve structure: *SHELXTL* (Sheldrick, 2008 ▶); program(s) used to refine structure: *SHELXTL* ; molecular graphics: *SHELXTL*; software used to prepare material for publication: *SHELXTL*.

## Supplementary Material

Crystal structure: contains datablocks global, I. DOI: 10.1107/S1600536810008780/fl2294sup1.cif

Structure factors: contains datablocks I. DOI: 10.1107/S1600536810008780/fl2294Isup2.hkl

Additional supplementary materials:  crystallographic information; 3D view; checkCIF report

## Figures and Tables

**Table 1:** Hydrogen-bond geometry (Å, °)

*D*—H⋯*A*	*D*—H	H⋯*A*	*D*⋯*A*	*D*—H⋯*A*
N1—H1⋯N4^i^	0.91	2.43	3.124 (2)	134
N2—H2*A*⋯O1	0.91	2.12	2.841 (2)	135
N2—H2*B*⋯N5^ii^	0.91	2.43	3.322 (2)	165
N3—H3⋯N2^i^	0.90	2.50	3.125 (2)	127
N4—H4*A*⋯O2	0.91	2.08	2.860 (2)	143
N4—H4*B*⋯O2^iii^	0.91	2.35	3.108 (2)	142
C32—H32*A*⋯O1	0.98	2.55	3.245 (2)	128

Symmetry codes: (i) 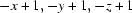; (ii) 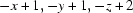; (iii) 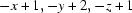.

**Fig. 1.**
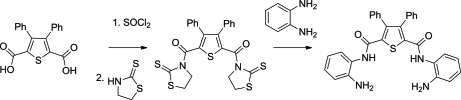
Synthesis of the ligand I.

**Fig. 2.**
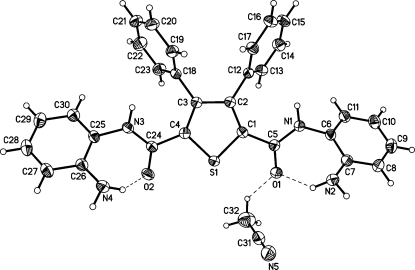
Molecular structure of I.CH3CN. Displacement ellipsoids are shown at the 50% probability level. Dashed lines indicate the hydrogen bonds.

**Fig. 3.**
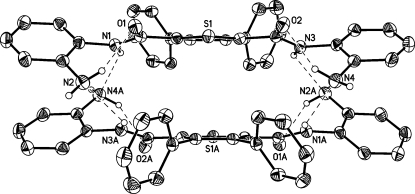
Centrosymmetrical dimers of I. Displacement ellipsoids are shown at the 50% probability level. Only H-atoms participating in the formation of the hydrogen bonds are presented. Dashed lines indicate the hydrogen bonds.

**Fig. 4.**
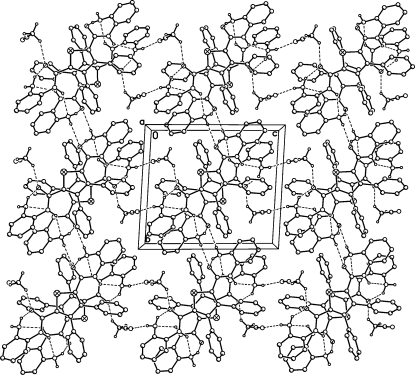
Crystal packing of dimers of I. Dashed lines indicate the hydrogen bonds.

## supplementary crystallographic information

### Comment

Aromatic diamide diamines are useful precursors for the construction of larger molecules which are used in  host-guest chemistry (Schneider &Yatsimirsky, 2008). Such diamines can bind neutral or anionic species using hydrogen bonds. We and others have reported several approaches for the synthesis of such type of diamines (Picard *et al.*, 2001; Sessler *et al.*, 2005*a*, 2005*b*; Katayev *et al.*, 2007). In this work we present the synthesis and crystal structure of a diamine used by us recently to prepare anion selective receptors (Sessler *et al.*, 2005*a*; Askerov *et al.*, 2010).

The synthesis consists of the conversion of dicarboxylic acid into the corresponding chloride followed by coupling with 2-mercaptothiazoline (Fig. 1). The activated acid was transformed into (I) by the reaction with 1,2-phenylenediamine.

(I) crystallizes as a solvate with an acetonitrile molecule. The molecule possesses approximate *C*_s_(m) intrinsic symmetry, with the mirror plane passing through the sulfur atom and the middle of the (Ph)C—C(Ph) bond (Fig. 2). Despite the main backbone of (I) being a long chain of conjugated bonds (C_Ar_—N(H)—C(O)—C═C—C(O)—N(H)—C_Ar_), it adopts a non-planar conformation due to the presence of various intra- and intermolecular hydrogen bonding interactions (Table 1). These hydrogen bonds result in  *twist* configurations for both the amido and aminophenyl fragments relative to the central thiophene ring. The dihedral angles between the O1═C5—N1—H1 and O2═ C24—N3—H3 amido planes and the S1—C1—C2—C3—C4 thiophene ring plane are 16.08 (8) and 19.30 (11)°, respectively, while that between the N2—C7—C8—C9—C10—C11—C6 and N4—C26—C27—C28—C29—C30—C25 aminophenyl planes and the S1—C1—C2—C3—C4 thiophene ring plane are 39.16 (5) and 33.43 (6)°, respectively. The dihedral angles between the planes of the C12—C13—C14—C15—C16—C17 and C18—C19—C20—C21—C22—C23 phenyl substituents and the S1—C1—C2—C3—C4 thiophene ring plane are 62.55 (6) and 74.62 (5)°, respectively.

There are two intramolecular N_amine_—H···O hydrogen bonds in (I) closing the O1—C5—N1—C6—C7—N2—H2A and O2—C24—N3—C25—C26—N4—H4A seven-membered rings (Table 1, Fig. 2). In the crystal, the molecules form centrosymmetrical dimers through four N1—H1···N4^i^ and N3—H3···N2^i^ hydrogen bonds (Table 1, Fig. 3). The dimers are further linked into layers parallel to (100) both directly (*via* N4—H4B···O2^iii^ hydrogen bonds, Table 1) and through the solvate acetonitrile molecules (*via* N2—H2B···N5^ii^ and C32—H32A···O1 hydrogen bonds, Table 1) (Fig. 4).

### Experimental

2,5-Bis((2-thio-1,3-thiazolidine-3-yl)carbonyl)-3,4-diphenylthiophene (II). 3,4-Diphenylthiophene-2,5-dicarboxylic acid (9 g, 27.7 mmol) was suspended in 35 ml freshly distilled SOCl_2_ in the presence of several drops of DMF. The resulting mixture was heated at reflux for 1 hour. The excess SOCl_2_ was removed under reduced pressure and the residue was further dried at 373 K under high vacuum. The thiophene diacid chloride obtained in this way was dissolved in 130 ml dry THF and added drop-wise during a 2 hour period to a solution containing 1,3-thiazolidine-2-thione (6.6 g, 55.4 mmol) and triethylamine (20 ml) in 330 ml dry THF. During this process carried out under continuous stirring the reaction temperature was kept at 323 K. After the addition was complete the reaction mixture was maintained under the same conditions for an additional 2 hours and then for a further 16 h at room temperature with stirring. The reaction mixture was then filtered, and the resulting solid was washed with cold THF. The filtrate was reduced in volume to a dark paste using a rotary evaporator. This paste-like material was then taken up into 30 ml of ethyl acetate. After this mixing procedure, crude product was filtered off as a dark-yellow powder. Recrystallization from dichloroethane yielded 10.8 g (74%) of yellow crystals. M.p. = 527-529 K. Found: C, 54.73; H, 3.44; N, 5.32. Calcd for C_24_H_18_N_2_O_2_S_5_: C, 54.98; H, 3.23; N, 5.36. ^1^H NMR (400 MHz, CDCl_3_): \d = 2.72 (t, 4H), 4.21 (t, 4H), 7.05 (m, 4H), 7.22 (m. 6H). ^13^C NMR (100 MHz, CDCl_3_): \d = 29.61, 55.80, 127.90, 127.99, 129.60, 134.21, 136.08, 144.34, 164.16, 200.37. Mass spectrometry (ESI+): 548.9 [*M+Na*]^+^, 1074.3 [2*M+Na*]^+^, 1601.8 [3*M+Na*]^+^.

(II) (5.0 g, 9.45 mmol) was added to a solution of 1,2-diaminobenzene (3.1 g, 28.4 mmol) in 125 ml of dry methylene chloride. The resulting mixture was stirred at room temperature for 2 days. At this juncture, the desired product, compound I, was obtained *via* filtration in a yield of 78% (3.7 g). M.p. = 501-503 K. Found: C, 71.40; H, 4.77; N, 11.45. Calcd for C_30_H_24_N_4_O_2_S: C, 71.41; H, 4.79; N, 11.10. ^1^H NMR (400 MHz, DMSO-*d*_6_): δ = 4.50 (s, 4H), 6.53 (t, 2H), 6.68 (d, 2H), 6.90 (t, 2H), 7.02 (d, 2H), 7.17 (m, 4H), 7.25 (m, 6H), 8.76 (s, 2H). ^13^C NMR (100 MHz, DMSO-*d*_6_): δ = 127.48, 127.90, 133.71, 136.90, 138.13, 139.58, 139.37, 141.32, 145.59, 147.12, 153.39, 153.48, 171.99. Mass spectrometry (ESI+): 527.1 [*M+Na*]^+^, 1030.9 [2*M+Na*]^+^, 1535.8 [3*M+Na*]^+^. Crystals suitable for X-ray diffraction were obtained by slow evaporation from an acetonitrile solution.

### Refinement

The hydrogen atoms of the amino-groups as well as the solvate acetonitrile molecule were localized in the difference-Fourier map and included in the refinement with fixed positional (C–H = 0.98 Å) and isotropic displacement parameters [*U*_iso_(H) = 1.5*U*_eq_(C) for CH_3_-group and *U*_iso_(H) = 1.2*U*_eq_(N) for amino groups]. The other hydrogen atoms were placed in calculated positions with C–H = 0.95 Å and refined in the riding model with fixed isotropic displacement parameters [*U*_iso_(H) = 1.2*U*_eq_(C)].

### Crystal data

**Table:**

C_30_H_24_N_4_O_2_S·C_2_H_3_N	*Z* = 2
*M**_r_* = 545.65	*F*(000) = 572
Triclinic, *P*1	*D*_x_ = 1.339 Mg m^−^^3^
Hall symbol: -P 1	Mo *K*α radiation, λ = 0.71073 Å
*a* = 9.0314 (9) Å	Cell parameters from 7103 reflections
*b* = 11.5470 (11) Å	θ = 2.3–28.0°
*c* = 13.0140 (12) Å	µ = 0.16 mm^−^^1^
α = 93.206 (2)°	*T* = 120 K
β = 92.504 (2)°	Prism, yellow
γ = 90.017 (2)°	0.24 × 0.21 × 0.18 mm
*V* = 1353.7 (2) Å^3^	

### Data collection

**Table:**

Bruker SMART 1K CCD diffractometer	6491 independent reflections
Radiation source: normal-focus sealed tube	5153 reflections with *I* > 2σ(*I*)
graphite	*R*_int_ = 0.018
φ and ω scans	θ_max_ = 28.1°, θ_min_ = 2.3°
Absorption correction: multi-scan (*SADABS*; Sheldrick, 1998)	*h* = −11→11
*T*_min_ = 0.965, *T*_max_ = 0.972	*k* = −15→15
13964 measured reflections	*l* = −16→17

### Refinement

**Table:**

Refinement on *F*^2^	Primary atom site location: structure-invariant direct methods
Least-squares matrix: full	Secondary atom site location: difference Fourier map
*R*[*F*^2^ > 2σ(*F*^2^)] = 0.048	Hydrogen site location: difference Fourier map
*wR*(*F*^2^) = 0.122	H-atom parameters constrained
*S* = 1.00	*w* = 1/[σ^2^(*F*_o_^2^) + (0.06*P*)^2^ + 0.82*P*] where *P* = (*F*_o_^2^ + 2*F*_c_^2^)/3
6491 reflections	(Δ/σ)_max_ = 0.001
362 parameters	Δρ_max_ = 0.40 e Å^−^^3^
0 restraints	Δρ_min_ = −0.26 e Å^−^^3^

### Special details

**Table:**

Geometry. All esds (except the esd in the dihedral angle between two l.s. planes) are estimated using the full covariance matrix. The cell esds are taken into account individually in the estimation of esds in distances, angles and torsion angles; correlations between esds in cell parameters are only used when they are defined by crystal symmetry. An approximate (isotropic) treatment of cell esds is used for estimating esds involving l.s. planes.
Refinement. Refinement of *F*^2^ against ALL reflections. The weighted *R*-factor wR and goodness of fit *S* are based on *F*^2^, conventional *R*-factors *R* are based on *F*, with *F* set to zero for negative *F*^2^. The threshold expression of *F*^2^ > σ(*F*^2^) is used only for calculating *R*-factors(gt) etc. and is not relevant to the choice of reflections for refinement. *R*-factors based on *F*^2^ are statistically about twice as large as those based on *F*, and *R*- factors based on ALL data will be even larger.

### Fractional atomic coordinates and isotropic or equivalent isotropic displacement parameters (Å^2^)

**Table:**

	*x*	*y*	*z*	*U*_iso_*/*U*_eq_	
S1	0.32334 (4)	0.62254 (3)	0.58968 (3)	0.02296 (11)	
O1	0.24008 (14)	0.49411 (10)	0.75610 (9)	0.0285 (3)	
O2	0.35642 (14)	0.83180 (10)	0.48613 (9)	0.0309 (3)	
N1	0.23655 (15)	0.31177 (11)	0.68036 (10)	0.0241 (3)	
H1	0.2481	0.2662	0.6220	0.029*	
N2	0.42197 (16)	0.33682 (12)	0.86449 (11)	0.0276 (3)	
H2A	0.4023	0.4114	0.8488	0.033*	
H2B	0.4786	0.3342	0.9242	0.033*	
N3	0.40740 (15)	0.75501 (11)	0.32722 (10)	0.0239 (3)	
H3	0.4154	0.6902	0.2858	0.029*	
N4	0.62869 (16)	0.91705 (12)	0.41468 (11)	0.0281 (3)	
H4A	0.5702	0.8894	0.4633	0.034*	
H4B	0.6792	0.9802	0.4416	0.034*	
C1	0.26943 (17)	0.47972 (13)	0.57739 (12)	0.0225 (3)	
C2	0.25071 (17)	0.43918 (13)	0.47550 (12)	0.0216 (3)	
C3	0.28338 (17)	0.52709 (13)	0.40610 (12)	0.0217 (3)	
C4	0.32623 (17)	0.63027 (14)	0.45839 (12)	0.0225 (3)	
C5	0.24737 (17)	0.42824 (14)	0.67855 (12)	0.0229 (3)	
C6	0.20591 (18)	0.25151 (13)	0.77046 (12)	0.0238 (3)	
C7	0.29838 (18)	0.26348 (14)	0.86012 (12)	0.0250 (3)	
C8	0.2672 (2)	0.19380 (15)	0.94138 (13)	0.0308 (4)	
H8	0.3280	0.1996	1.0029	0.037*	
C9	0.1493 (2)	0.11687 (16)	0.93351 (14)	0.0346 (4)	
H9	0.1306	0.0703	0.9895	0.042*	
C10	0.0585 (2)	0.10680 (16)	0.84531 (15)	0.0336 (4)	
H10	−0.0227	0.0541	0.8404	0.040*	
C11	0.08769 (19)	0.17493 (14)	0.76388 (13)	0.0283 (3)	
H11	0.0257	0.1688	0.7029	0.034*	
C12	0.19053 (17)	0.32216 (13)	0.44120 (12)	0.0221 (3)	
C13	0.04857 (18)	0.29170 (14)	0.46735 (13)	0.0263 (3)	
H13	−0.0102	0.3464	0.5045	0.032*	
C14	−0.0081 (2)	0.18195 (15)	0.43950 (14)	0.0314 (4)	
H14	−0.1044	0.1614	0.4589	0.038*	
C15	0.0755 (2)	0.10268 (15)	0.38373 (14)	0.0323 (4)	
H15	0.0366	0.0276	0.3651	0.039*	
C16	0.2158 (2)	0.13241 (15)	0.35487 (14)	0.0306 (4)	
H16	0.2724	0.0784	0.3154	0.037*	
C17	0.27350 (19)	0.24181 (14)	0.38395 (13)	0.0269 (3)	
H17	0.3701	0.2619	0.3647	0.032*	
C18	0.25880 (17)	0.51370 (13)	0.29169 (12)	0.0225 (3)	
C19	0.3573 (2)	0.45298 (15)	0.22964 (13)	0.0282 (3)	
H19	0.4423	0.4177	0.2600	0.034*	
C20	0.3317 (2)	0.44371 (16)	0.12320 (14)	0.0343 (4)	
H20	0.3991	0.4015	0.0814	0.041*	
C21	0.2092 (2)	0.49542 (17)	0.07780 (14)	0.0360 (4)	
H21	0.1922	0.4888	0.0051	0.043*	
C22	0.1116 (2)	0.55669 (18)	0.13886 (15)	0.0383 (4)	
H22	0.0277	0.5928	0.1080	0.046*	
C23	0.1357 (2)	0.56590 (16)	0.24561 (13)	0.0307 (4)	
H23	0.0679	0.6080	0.2871	0.037*	
C24	0.36380 (17)	0.74725 (13)	0.42408 (12)	0.0229 (3)	
C25	0.43963 (18)	0.86137 (13)	0.28064 (12)	0.0240 (3)	
C26	0.54734 (18)	0.93901 (14)	0.32371 (13)	0.0249 (3)	
C27	0.5790 (2)	1.03668 (15)	0.26837 (14)	0.0319 (4)	
H27	0.6484	1.0927	0.2970	0.038*	
C28	0.5105 (2)	1.05222 (16)	0.17287 (15)	0.0363 (4)	
H28	0.5352	1.1178	0.1360	0.044*	
C29	0.4066 (2)	0.97321 (16)	0.13054 (14)	0.0346 (4)	
H29	0.3604	0.9839	0.0647	0.041*	
C30	0.37053 (19)	0.87819 (15)	0.18545 (13)	0.0282 (3)	
H30	0.2979	0.8243	0.1576	0.034*	
N5	0.3348 (2)	0.71107 (17)	0.94378 (15)	0.0518 (5)	
C31	0.2246 (3)	0.71979 (17)	0.89885 (16)	0.0402 (5)	
C32	0.0842 (3)	0.7313 (2)	0.8419 (2)	0.0555 (6)	
H32A	0.0772	0.6723	0.7847	0.083*	
H32B	0.0027	0.7209	0.8878	0.083*	
H32C	0.0778	0.8087	0.8146	0.083*	

### Atomic displacement parameters (Å^2^)

**Table:**

	*U*^11^	*U*^22^	*U*^33^	*U*^12^	*U*^13^	*U*^23^
S1	0.0254 (2)	0.02091 (19)	0.02226 (19)	−0.00233 (14)	0.00057 (14)	−0.00059 (14)
O1	0.0368 (7)	0.0234 (6)	0.0250 (6)	0.0031 (5)	0.0028 (5)	−0.0015 (4)
O2	0.0380 (7)	0.0236 (6)	0.0313 (6)	−0.0048 (5)	0.0103 (5)	−0.0040 (5)
N1	0.0285 (7)	0.0221 (6)	0.0218 (6)	0.0015 (5)	0.0010 (5)	0.0006 (5)
N2	0.0301 (7)	0.0267 (7)	0.0255 (7)	−0.0006 (6)	−0.0023 (6)	0.0002 (5)
N3	0.0270 (7)	0.0192 (6)	0.0254 (7)	−0.0022 (5)	0.0024 (5)	−0.0008 (5)
N4	0.0283 (7)	0.0262 (7)	0.0294 (7)	−0.0042 (6)	−0.0005 (6)	−0.0009 (6)
C1	0.0207 (7)	0.0202 (7)	0.0264 (8)	0.0001 (6)	0.0002 (6)	0.0016 (6)
C2	0.0192 (7)	0.0215 (7)	0.0239 (7)	0.0014 (6)	0.0006 (6)	−0.0001 (6)
C3	0.0202 (7)	0.0217 (7)	0.0232 (7)	0.0011 (6)	0.0008 (6)	0.0006 (6)
C4	0.0206 (7)	0.0237 (7)	0.0233 (7)	−0.0002 (6)	0.0017 (6)	0.0009 (6)
C5	0.0216 (7)	0.0231 (7)	0.0240 (7)	0.0019 (6)	0.0010 (6)	0.0017 (6)
C6	0.0282 (8)	0.0202 (7)	0.0235 (7)	0.0029 (6)	0.0038 (6)	0.0018 (6)
C7	0.0260 (8)	0.0238 (8)	0.0253 (8)	0.0036 (6)	0.0025 (6)	0.0000 (6)
C8	0.0378 (10)	0.0308 (9)	0.0241 (8)	0.0021 (7)	−0.0007 (7)	0.0043 (7)
C9	0.0394 (10)	0.0331 (9)	0.0328 (9)	0.0003 (8)	0.0064 (8)	0.0106 (7)
C10	0.0309 (9)	0.0301 (9)	0.0405 (10)	−0.0044 (7)	0.0023 (8)	0.0075 (7)
C11	0.0283 (8)	0.0245 (8)	0.0319 (9)	−0.0006 (6)	−0.0026 (7)	0.0032 (6)
C12	0.0241 (8)	0.0215 (7)	0.0205 (7)	−0.0014 (6)	−0.0019 (6)	0.0018 (6)
C13	0.0240 (8)	0.0267 (8)	0.0277 (8)	0.0007 (6)	0.0006 (6)	−0.0023 (6)
C14	0.0258 (8)	0.0319 (9)	0.0361 (9)	−0.0082 (7)	0.0003 (7)	0.0006 (7)
C15	0.0370 (10)	0.0220 (8)	0.0371 (9)	−0.0073 (7)	−0.0031 (7)	−0.0020 (7)
C16	0.0327 (9)	0.0247 (8)	0.0336 (9)	0.0025 (7)	0.0013 (7)	−0.0043 (7)
C17	0.0255 (8)	0.0245 (8)	0.0308 (8)	−0.0005 (6)	0.0040 (6)	0.0009 (6)
C18	0.0242 (8)	0.0205 (7)	0.0226 (7)	−0.0051 (6)	−0.0007 (6)	0.0008 (6)
C19	0.0294 (9)	0.0286 (8)	0.0266 (8)	0.0008 (7)	0.0016 (6)	−0.0002 (6)
C20	0.0403 (10)	0.0357 (9)	0.0264 (9)	−0.0049 (8)	0.0054 (7)	−0.0052 (7)
C21	0.0464 (11)	0.0377 (10)	0.0232 (8)	−0.0113 (8)	−0.0027 (7)	−0.0005 (7)
C22	0.0375 (10)	0.0440 (11)	0.0325 (9)	−0.0004 (8)	−0.0099 (8)	0.0044 (8)
C23	0.0298 (9)	0.0330 (9)	0.0287 (9)	0.0030 (7)	−0.0011 (7)	−0.0006 (7)
C24	0.0222 (7)	0.0208 (7)	0.0258 (8)	−0.0011 (6)	0.0024 (6)	0.0000 (6)
C25	0.0248 (8)	0.0206 (7)	0.0272 (8)	0.0007 (6)	0.0056 (6)	0.0020 (6)
C26	0.0248 (8)	0.0217 (7)	0.0282 (8)	0.0016 (6)	0.0049 (6)	−0.0002 (6)
C27	0.0309 (9)	0.0256 (8)	0.0397 (10)	−0.0046 (7)	0.0071 (7)	0.0029 (7)
C28	0.0400 (10)	0.0297 (9)	0.0408 (10)	−0.0012 (8)	0.0075 (8)	0.0121 (8)
C29	0.0366 (10)	0.0361 (10)	0.0319 (9)	0.0040 (8)	0.0021 (7)	0.0097 (7)
C30	0.0275 (8)	0.0273 (8)	0.0297 (8)	0.0010 (7)	0.0019 (7)	0.0014 (7)
N5	0.0561 (12)	0.0509 (11)	0.0484 (11)	−0.0142 (9)	−0.0086 (9)	0.0104 (9)
C31	0.0526 (13)	0.0312 (10)	0.0372 (10)	−0.0072 (9)	0.0043 (9)	0.0038 (8)
C32	0.0540 (14)	0.0523 (14)	0.0587 (15)	0.0085 (11)	−0.0028 (11)	−0.0056 (11)

### Geometric parameters (Å, °)

**Table:**

S1—C1	1.7162 (16)	C13—C14	1.390 (2)
S1—C4	1.7171 (16)	C13—H13	0.9500
O1—C5	1.2334 (19)	C14—C15	1.381 (3)
O2—C24	1.2354 (19)	C14—H14	0.9500
N1—C5	1.350 (2)	C15—C16	1.386 (3)
N1—C6	1.434 (2)	C15—H15	0.9500
N1—H1	0.9092	C16—C17	1.393 (2)
N2—C7	1.398 (2)	C16—H16	0.9500
N2—H2A	0.9120	C17—H17	0.9500
N2—H2B	0.9136	C18—C19	1.391 (2)
N3—C24	1.345 (2)	C18—C23	1.393 (2)
N3—C25	1.435 (2)	C19—C20	1.392 (2)
N3—H3	0.9029	C19—H19	0.9500
N4—C26	1.401 (2)	C20—C21	1.383 (3)
N4—H4A	0.9125	C20—H20	0.9500
N4—H4B	0.9060	C21—C22	1.381 (3)
C1—C2	1.384 (2)	C21—H21	0.9500
C1—C5	1.495 (2)	C22—C23	1.395 (2)
C2—C3	1.435 (2)	C22—H22	0.9500
C2—C12	1.494 (2)	C23—H23	0.9500
C3—C4	1.385 (2)	C25—C30	1.386 (2)
C3—C18	1.495 (2)	C25—C26	1.400 (2)
C4—C24	1.490 (2)	C26—C27	1.407 (2)
C6—C11	1.383 (2)	C27—C28	1.385 (3)
C6—C7	1.405 (2)	C27—H27	0.9500
C7—C8	1.402 (2)	C28—C29	1.384 (3)
C8—C9	1.383 (3)	C28—H28	0.9500
C8—H8	0.9500	C29—C30	1.389 (2)
C9—C10	1.381 (3)	C29—H29	0.9500
C9—H9	0.9500	C30—H30	0.9500
C10—C11	1.389 (2)	N5—C31	1.139 (3)
C10—H10	0.9500	C31—C32	1.451 (3)
C11—H11	0.9500	C32—H32A	0.9800
C12—C13	1.391 (2)	C32—H32B	0.9800
C12—C17	1.396 (2)	C32—H32C	0.9800
			
C1—S1—C4	91.55 (8)	C14—C15—C16	120.17 (16)
C5—N1—C6	124.09 (13)	C14—C15—H15	119.9
C5—N1—H1	120.3	C16—C15—H15	119.9
C6—N1—H1	115.6	C15—C16—C17	119.79 (16)
C7—N2—H2A	114.9	C15—C16—H16	120.1
C7—N2—H2B	113.7	C17—C16—H16	120.1
H2A—N2—H2B	111.3	C16—C17—C12	120.48 (16)
C24—N3—C25	124.93 (13)	C16—C17—H17	119.8
C24—N3—H3	119.9	C12—C17—H17	119.8
C25—N3—H3	115.2	C19—C18—C23	119.01 (15)
C26—N4—H4A	112.0	C19—C18—C3	121.86 (15)
C26—N4—H4B	112.6	C23—C18—C3	119.11 (14)
H4A—N4—H4B	109.5	C18—C19—C20	120.21 (16)
C2—C1—C5	134.26 (14)	C18—C19—H19	119.9
C2—C1—S1	112.53 (12)	C20—C19—H19	119.9
C5—C1—S1	113.16 (11)	C21—C20—C19	120.58 (17)
C1—C2—C3	111.71 (14)	C21—C20—H20	119.7
C1—C2—C12	124.44 (14)	C19—C20—H20	119.7
C3—C2—C12	123.63 (14)	C22—C21—C20	119.51 (17)
C4—C3—C2	111.76 (14)	C22—C21—H21	120.2
C4—C3—C18	123.61 (14)	C20—C21—H21	120.2
C2—C3—C18	124.32 (14)	C21—C22—C23	120.36 (18)
C3—C4—C24	133.19 (14)	C21—C22—H22	119.8
C3—C4—S1	112.44 (12)	C23—C22—H22	119.8
C24—C4—S1	114.22 (11)	C18—C23—C22	120.32 (17)
O1—C5—N1	123.27 (15)	C18—C23—H23	119.8
O1—C5—C1	118.49 (14)	C22—C23—H23	119.8
N1—C5—C1	118.25 (14)	O2—C24—N3	123.20 (15)
C11—C6—C7	120.89 (15)	O2—C24—C4	118.76 (14)
C11—C6—N1	117.59 (15)	N3—C24—C4	118.02 (14)
C7—C6—N1	121.37 (15)	C30—C25—C26	121.14 (15)
N2—C7—C8	121.83 (15)	C30—C25—N3	116.87 (14)
N2—C7—C6	120.62 (15)	C26—C25—N3	121.66 (15)
C8—C7—C6	117.42 (15)	C25—C26—N4	121.71 (15)
C9—C8—C7	121.12 (16)	C25—C26—C27	117.49 (16)
C9—C8—H8	119.4	N4—C26—C27	120.64 (15)
C7—C8—H8	119.4	C28—C27—C26	120.95 (16)
C10—C9—C8	120.84 (17)	C28—C27—H27	119.5
C10—C9—H9	119.6	C26—C27—H27	119.5
C8—C9—H9	119.6	C29—C28—C27	120.68 (17)
C9—C10—C11	118.92 (17)	C29—C28—H28	119.7
C9—C10—H10	120.5	C27—C28—H28	119.7
C11—C10—H10	120.5	C28—C29—C30	119.16 (17)
C6—C11—C10	120.80 (16)	C28—C29—H29	120.4
C6—C11—H11	119.6	C30—C29—H29	120.4
C10—C11—H11	119.6	C25—C30—C29	120.52 (16)
C13—C12—C17	118.89 (15)	C25—C30—H30	119.7
C13—C12—C2	119.39 (14)	C29—C30—H30	119.7
C17—C12—C2	121.72 (14)	N5—C31—C32	179.8 (3)
C14—C13—C12	120.54 (16)	C31—C32—H32A	109.5
C14—C13—H13	119.7	C31—C32—H32B	109.5
C12—C13—H13	119.7	H32A—C32—H32B	109.5
C15—C14—C13	120.11 (16)	C31—C32—H32C	109.5
C15—C14—H14	119.9	H32A—C32—H32C	109.5
C13—C14—H14	119.9	H32B—C32—H32C	109.5
			
C4—S1—C1—C2	−1.32 (13)	C17—C12—C13—C14	1.8 (2)
C4—S1—C1—C5	−179.05 (12)	C2—C12—C13—C14	−177.74 (15)
C5—C1—C2—C3	177.89 (16)	C12—C13—C14—C15	−1.2 (3)
S1—C1—C2—C3	0.80 (17)	C13—C14—C15—C16	−0.3 (3)
C5—C1—C2—C12	3.2 (3)	C14—C15—C16—C17	1.2 (3)
S1—C1—C2—C12	−173.88 (12)	C15—C16—C17—C12	−0.6 (3)
C1—C2—C3—C4	0.33 (19)	C13—C12—C17—C16	−0.9 (2)
C12—C2—C3—C4	175.06 (14)	C2—C12—C17—C16	178.63 (15)
C1—C2—C3—C18	−173.50 (14)	C4—C3—C18—C19	107.51 (19)
C12—C2—C3—C18	1.2 (2)	C2—C3—C18—C19	−79.4 (2)
C2—C3—C4—C24	−176.42 (16)	C4—C3—C18—C23	−70.9 (2)
C18—C3—C4—C24	−2.5 (3)	C2—C3—C18—C23	102.24 (19)
C2—C3—C4—S1	−1.32 (17)	C23—C18—C19—C20	−0.7 (3)
C18—C3—C4—S1	172.56 (12)	C3—C18—C19—C20	−179.11 (15)
C1—S1—C4—C3	1.51 (13)	C18—C19—C20—C21	0.6 (3)
C1—S1—C4—C24	177.59 (12)	C19—C20—C21—C22	0.0 (3)
C6—N1—C5—O1	4.2 (3)	C20—C21—C22—C23	−0.4 (3)
C6—N1—C5—C1	−175.83 (14)	C19—C18—C23—C22	0.3 (3)
C2—C1—C5—O1	−163.05 (17)	C3—C18—C23—C22	178.76 (16)
S1—C1—C5—O1	14.02 (19)	C21—C22—C23—C18	0.2 (3)
C2—C1—C5—N1	17.0 (3)	C25—N3—C24—O2	−5.1 (3)
S1—C1—C5—N1	−165.92 (12)	C25—N3—C24—C4	176.44 (14)
C5—N1—C6—C11	125.82 (17)	C3—C4—C24—O2	158.95 (17)
C5—N1—C6—C7	−58.5 (2)	S1—C4—C24—O2	−16.1 (2)
C11—C6—C7—N2	176.85 (15)	C3—C4—C24—N3	−22.5 (3)
N1—C6—C7—N2	1.3 (2)	S1—C4—C24—N3	162.48 (12)
C11—C6—C7—C8	0.9 (2)	C24—N3—C25—C30	−128.99 (17)
N1—C6—C7—C8	−174.68 (14)	C24—N3—C25—C26	57.6 (2)
N2—C7—C8—C9	−176.27 (16)	C30—C25—C26—N4	−173.44 (15)
C6—C7—C8—C9	−0.4 (3)	N3—C25—C26—N4	−0.3 (2)
C7—C8—C9—C10	−0.3 (3)	C30—C25—C26—C27	2.1 (2)
C8—C9—C10—C11	0.4 (3)	N3—C25—C26—C27	175.29 (15)
C7—C6—C11—C10	−0.8 (3)	C25—C26—C27—C28	−2.8 (3)
N1—C6—C11—C10	174.92 (15)	N4—C26—C27—C28	172.85 (16)
C9—C10—C11—C6	0.2 (3)	C26—C27—C28—C29	1.5 (3)
C1—C2—C12—C13	59.9 (2)	C27—C28—C29—C30	0.5 (3)
C3—C2—C12—C13	−114.13 (18)	C26—C25—C30—C29	−0.2 (3)
C1—C2—C12—C17	−119.63 (18)	N3—C25—C30—C29	−173.68 (15)
C3—C2—C12—C17	66.3 (2)	C28—C29—C30—C25	−1.2 (3)

### Hydrogen-bond geometry (Å, °)

**Table:**

*D*—H···*A*	*D*—H	H···*A*	*D*···*A*	*D*—H···*A*
N1—H1···N4^i^	0.91	2.43	3.124 (2)	134
N2—H2A···O1	0.91	2.12	2.841 (2)	135
N2—H2B···N5^ii^	0.91	2.43	3.322 (2)	165
N3—H3···N2^i^	0.90	2.50	3.125 (2)	127
N4—H4A···O2	0.91	2.08	2.860 (2)	143
N4—H4B···O2^iii^	0.91	2.35	3.108 (2)	142
C32—H32A···O1	0.98	2.55	3.245 (2)	128

Symmetry codes: (i) −*x*+1, −*y*+1, −*z*+1; (ii) −*x*+1, −*y*+1, −*z*+2; (iii) −*x*+1, −*y*+2, −*z*+1.
